# *catena*-Poly[[[triaqua­manganese(II)]-μ-4,4′-bipyridine-κ^2^ *N*:*N*′-[triaqua­manganese(II)]-μ-pyrimidine-4,6-dicarboxyl­ato-κ^4^ *N* ^1^,*O* ^6^:*N* ^3^,*O* ^4^] sulfate trihydrate]

**DOI:** 10.1107/S1600536809053896

**Published:** 2009-12-19

**Authors:** Wenguo Wang, Elisa Barea, Fátima Linares

**Affiliations:** a Departamento de Química Inorgánica, Facultad de Ciencias, Universidad de Granada, Avda. Fuentenueva s/n, 18071 Granada, Spain

## Abstract

The two independent Mn^II^ ions in the polymeric title compound, {[Mn_2_(C_6_H_2_N_2_O_4_)(C_10_H_8_N_2_)(H_2_O)_6_]SO_4_·3H_2_O}, exhibit distorted MnN_2_O_4_ octa­hedral coordination geometries, with the pyrimidine-4,6-dicarboxyl­ate (pmdc) ligand acting in the bis-chelating μ-(κ*O*,κ*N*:κ*O*′,κ*N*′) bridging mode and the 4,4′-bipyridine (bpy) ligand in the μ-(κ*N*:κ*N*′) bridging mode. The remaining coordination sites are occupied by O atoms of water mol­ecules. As a consequence, cationic chains of [Mn_2_(μ-pmdc)(μ-4,4′-bpy)(H_2_O)_6_]^2+^ are generated, which extend approximately along the *a* axis. Sulfate counter-anions and three uncoordinated water mol­ecules complete the structure, which is stabilized by multiple O—H⋯O hydrogen-bonding inter­actions between the structural units.

## Related literature

For the preparation of the pyrimidine-4,6-dicarboxyl­ato ligand (pmdc) we utilized the commercially available 4,6-dimethyl-pyrimidine, which can easily be oxidized to the corresponding dicarboxylic acid (H_2_pmdc), originally prepared by Hunt *et al.* (1959 ▶). For pmdc coordination compounds, see: Beobide *et al.* (2008 ▶); Masciocchi *et al.* (2009 ▶).
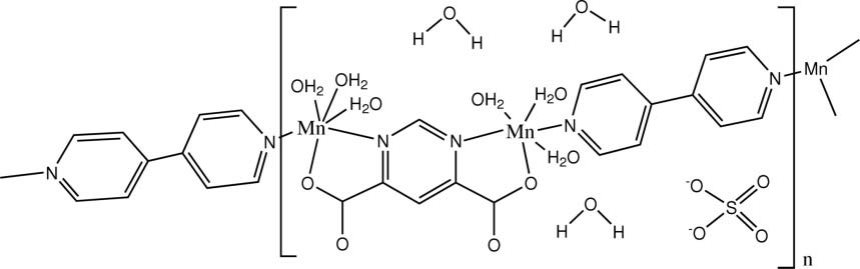


## Experimental

### 

#### Crystal data

[Mn_2_(C_6_H_2_N_2_O_4_)(C_10_H_8_N_2_)(H_2_O)_6_]SO_4_·3H_2_O
*M* *_r_* = 690.36
Monoclinic, 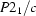

*a* = 18.745 (2) Å
*b* = 10.7639 (14) Å
*c* = 14.1585 (18) Å
β = 111.044 (2)°
*V* = 2666.2 (6) Å^3^
*Z* = 4
Mo *K*α radiation
μ = 1.11 mm^−1^
*T* = 298 K
0.32 × 0.27 × 0.21 mm

#### Data collection

Bruker SMART APEX CCD area-detector diffractometer
Absorption correction: multi-scan (*SADABS*; Sheldrick, 2004 ▶) *T*_min_ = 0.693, *T*_max_ = 0.794
30075 measured reflections
6219 independent reflections
5467 reflections with *I* > 2σ(*I*)
*R*_int_ = 0.029

#### Refinement

*R*[*F* ^2^ > 2σ(*F* ^2^)] = 0.041
*wR*(*F* ^2^) = 0.115
*S* = 1.09
6219 reflections
403 parameters
14 restraints
H atoms treated by a mixture of independent and constrained refinement
Δρ_max_ = 0.62 e Å^−3^
Δρ_min_ = −0.36 e Å^−3^

### 

Data collection: *SMART* (Bruker, 2001 ▶); cell refinement: *SAINT* (Bruker, 2001 ▶); data reduction: *SAINT*; program(s) used to solve structure: *SHELXTL* (Sheldrick, 2008 ▶); program(s) used to refine structure: *SHELXTL*; molecular graphics: *XP* in *SHELXTL*; software used to prepare material for publication: *publCIF* (Westrip, 2010 ▶).

## Supplementary Material

Crystal structure: contains datablocks global, I. DOI: 10.1107/S1600536809053896/wm2288sup1.cif

Structure factors: contains datablocks I. DOI: 10.1107/S1600536809053896/wm2288Isup2.hkl

Additional supplementary materials:  crystallographic information; 3D view; checkCIF report

## Figures and Tables

**Table 1:** Selected bond lengths (Å)

Mn1—O12*W*	2.174 (2)
Mn1—O11*W*	2.180 (2)
Mn1—O1*W*	2.187 (2)
Mn1—O42	2.188 (2)
Mn1—N1*B*^i^	2.219 (2)
Mn1—N3	2.272 (2)
Mn2—O2*W*	2.154 (2)
Mn2—O21*W*	2.170 (2)
Mn2—O22*W*	2.201 (2)
Mn2—O62	2.2055 (19)
Mn2—N1*B*1	2.214 (2)
Mn2—N1	2.282 (2)

Symmetry code: (i) 
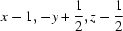
.

**Table 2:** Hydrogen-bond geometry (Å, °)

*D*—H⋯*A*	*D*—H	H⋯*A*	*D*⋯*A*	*D*—H⋯*A*
O1*W*—H1*AW*⋯O4*W*	0.82 (1)	1.85 (1)	2.667 (5)	176 (4)
O1*W*—H1*BW*⋯O2*S*^ii^	0.82 (1)	2.11 (2)	2.891 (3)	159 (4)
O2*W*—H2*BW*⋯O62^iii^	0.82 (1)	1.96 (1)	2.775 (3)	172 (4)
O2*W*—H2*AW*⋯O1*S*^ii^	0.82 (1)	1.87 (1)	2.681 (3)	172 (4)
O21*W*—H21*A*⋯O3*S*^iv^	0.82 (1)	1.86 (1)	2.663 (3)	166 (4)
O21*W*—H21*B*⋯O5*W*	0.82 (1)	1.97 (1)	2.782 (4)	172 (4)
O11*W*—H11*A*⋯O42^v^	0.82 (1)	1.95 (1)	2.760 (3)	167 (4)
O11*W*—H11*B*⋯O2*S*^iv^	0.82 (1)	1.99 (1)	2.797 (3)	168 (4)
O5*W*—H5*AW*⋯O4*S*^ii^	0.82	2.12	2.927 (4)	168
O5*W*—H5*BW*⋯O61^vi^	0.82	2.15	2.910 (3)	154
O4*W*—H4*AW*⋯O41^vii^	0.82 (1)	1.89 (1)	2.702 (4)	170 (6)
O4*W*—H4*BW*⋯O4*W*^viii^	0.82 (1)	2.48 (6)	2.908 (7)	114 (6)
O3*W*—H3*BW*⋯O3*S*^iv^	0.82	1.93	2.746 (5)	178
O12*W*—H12*A*⋯O3*W*	0.82 (1)	1.85 (1)	2.656 (4)	167 (4)
O12*W*—H12*B*⋯O2*S*^ii^	0.82 (1)	2.04 (1)	2.840 (3)	166 (4)
O22*W*—H22*A*⋯O4*S*^iv^	0.82 (1)	1.95 (1)	2.764 (3)	171 (3)
O22*W*—H22*B*⋯O61^ix^	0.82 (1)	2.03 (1)	2.852 (3)	177 (3)

Symmetry codes: (ii) 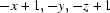; (iii) 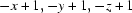; (iv) 
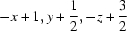
; (v) 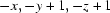; (vi) 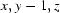; (vii) 
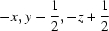
; (viii) 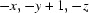; (ix) 
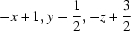
.

**Fig. 1.**
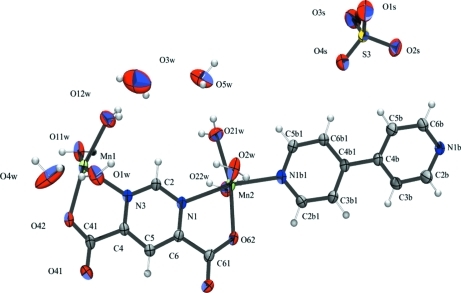
The asymmetric unit of the title compound. Thermal displacement parameters are drawn at the 50% probability level.

**Fig. 2.**
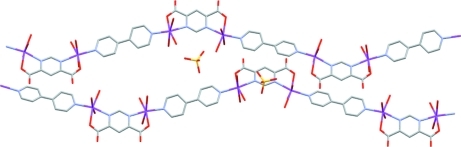
View of the crystal packing showing the formation of [Mn2(µ-4,4'bpy)(µ-pmdc)(H2O)6] chains interacting through multiple H-bonding with sulfate and uncoordinated water molecules.

## supplementary crystallographic information

### Comment

In the structure of the title compound there are two independent manganese ions. Each metal ion exhibits a distorted octahedral coordination geometry built up of one oxygen atom and one nitrogen atom from the bis-chelating pyrimidine-4,6-dicarboxylato ligand (pmdc), one nitrogen atom from the 4,4'-bipyridine (bpy) ligand and three oxygen atoms belonging to three coordinated water molecules (Fig. 1). The bridging nature of the pmdc and bpy ligands yields 1_D polymeric chains (Fig. 2) extending approximately along the *a* axis. The pmdc ligands adopt a µ-(κ*O*,κ*N*:κ*O*'',κ*N*') coordination mode, and their *N,O* chelation results in the formation of two five-membered chelate rings for each metal ion.

The pmdc ligand combines the *N,N*'-coordination features of pyrimidine to the donor properties of the carboxylate group. Moreover, possessing two easily removable acidic hydrogen atoms, it can be coupled to the *M*(II) ions of the transition metal series, in search for homoleptic coordination compounds of [*M*(pmdc)] formulation. In this communication, we have employed 4,4'-bipyridine ligand in order to have a further connectivity.

The structur is in agreement with previous crystallographic studies carried out in our group revealing that the pmdc ligand typically displays a tetradentate µ-(κ*O*,κ*N*:κ*O*',κ*N*') coordination mode with the carboxylate groups almost coplanar with the pyrimidine ring.

### Experimental

The ligand pyrimidine-4,6-dicarboxylic acid (H_2_pmdc) was prepared from the oxidation of 4,6-dimethyl-pyrimidine (Hunt *et al.*, 1959). The new metal complex [Mn_2_(µ-4,4'bpy)(µ-pmdc)(H_2_O)_6_]SO_4_3H_2_O was obtained by reaction of an aqueous solution (30 ml) containing pyrimidine-4,6-dicarboxylato (168.3 mg) and MnSO_4_(H_2_O) (169.0 mg) at 353 K during 4 h. The resulting yellow suspension was cooled to room temperature and filtered. Subsequent diffusion of 4,4'_bipyridine (312.4 mg), dissolved in 10 ml of methanol, into this solution yielded pale yellow crystals suitable for X-ray diffraction after two weeks.

### Refinement

The water H atoms were located in difference maps and were refined as riding with O—H = 0.82 Å and with *U*_iso_(H) = 1.2*U*_eq_(O). The pyrimidine an bipyridine H atoms were positioned geometrically and treated as riding with C—H = 0.93 Å, and with *U*_iso_(H) = 1.2*U*_eq_(C).

### Crystal data

**Table:**

[Mn_2_(C_6_H_2_N_2_O_4_)(C_10_H_8_N_2_)(H_2_O)_6_]SO_4_·3H_2_O	*F*(000) = 1416
*M**_r_* = 690.36	*D*_x_ = 1.720 Mg m^−^^3^
Monoclinic, *P*2_1_/*c*	Mo *K*α radiation, λ = 0.71073 Å
Hall symbol: -P 2ybc	Cell parameters from 5467 reflections
*a* = 18.745 (2) Å	θ = 2.2–28.3°
*b* = 10.7639 (14) Å	µ = 1.11 mm^−^^1^
*c* = 14.1585 (18) Å	*T* = 298 K
β = 111.044 (2)°	Prismatic, yellow
*V* = 2666.2 (6) Å^3^	0.32 × 0.27 × 0.21 mm
*Z* = 4	

### Data collection

**Table:**

Bruker SMART APEX CCD area-detector diffractometer	6219 independent reflections
Radiation source: fine-focus sealed tube	5467 reflections with *I* > 2σ(*I*)
graphite	*R*_int_ = 0.029
φ and ω scans	θ_max_ = 28.3°, θ_min_ = 2.2°
Absorption correction: multi-scan (*SADABS*; Sheldrick, 2004)	*h* = −24→24
*T*_min_ = 0.693, *T*_max_ = 0.794	*k* = −14→14
30075 measured reflections	*l* = −18→18

### Refinement

**Table:**

Refinement on *F*^2^	Primary atom site location: structure-invariant direct methods
Least-squares matrix: full	Secondary atom site location: difference Fourier map
*R*[*F*^2^ > 2σ(*F*^2^)] = 0.041	Hydrogen site location: inferred from neighbouring sites
*wR*(*F*^2^) = 0.115	H atoms treated by a mixture of independent and constrained refinement
*S* = 1.09	*w* = 1/[σ^2^(*F*_o_^2^) + (0.0452*P*)^2^ + 3.6924*P*] where *P* = (*F*_o_^2^ + 2*F*_c_^2^)/3
6219 reflections	(Δ/σ)_max_ = 0.001
403 parameters	Δρ_max_ = 0.62 e Å^−^^3^
14 restraints	Δρ_min_ = −0.36 e Å^−^^3^

### Special details

**Table:**

Geometry. All e.s.d.'s (except the e.s.d. in the dihedral angle between two l.s. planes) are estimated using the full covariance matrix. The cell e.s.d.'s are taken into account individually in the estimation of e.s.d.'s in distances, angles and torsion angles; correlations between e.s.d.'s in cell parameters are only used when they are defined by crystal symmetry. An approximate (isotropic) treatment of cell e.s.d.'s is used for estimating e.s.d.'s involving l.s. planes.
Refinement. Refinement of *F*^2^ against ALL reflections. The weighted *R*-factor *wR* and goodness of fit *S* are based on *F*^2^, conventional *R*-factors *R* are based on *F*, with *F* set to zero for negative *F*^2^. The threshold expression of *F*^2^ > σ(*F*^2^) is used only for calculating *R*-factors(gt) *etc*. and is not relevant to the choice of reflections for refinement. *R*-factors based on *F*^2^ are statistically about twice as large as those based on *F*, and *R*- factors based on ALL data will be even larger.

### Fractional atomic coordinates and isotropic or equivalent isotropic displacement parameters (Å^2^)

**Table:**

	*x*	*y*	*z*	*U*_iso_*/*U*_eq_	
Mn1	0.08050 (2)	0.39447 (4)	0.41167 (3)	0.02939 (11)	
Mn2	0.44821 (2)	0.39042 (4)	0.63222 (3)	0.02798 (11)	
S3	0.73306 (3)	−0.23319 (6)	0.71958 (5)	0.03205 (15)	
O42	0.06099 (10)	0.59414 (18)	0.41849 (16)	0.0379 (4)	
O62	0.46593 (10)	0.59002 (17)	0.61315 (15)	0.0347 (4)	
O61	0.41374 (11)	0.77834 (18)	0.57634 (16)	0.0402 (5)	
C6	0.33123 (13)	0.6050 (2)	0.55476 (18)	0.0254 (5)	
C4	0.19647 (13)	0.6050 (2)	0.48627 (19)	0.0269 (5)	
O41	0.11712 (12)	0.7803 (2)	0.4390 (2)	0.0518 (6)	
N3	0.19627 (11)	0.48139 (19)	0.49708 (16)	0.0282 (4)	
C4B1	0.72311 (13)	0.2647 (2)	0.73258 (18)	0.0275 (5)	
O4S	0.68705 (13)	−0.1207 (2)	0.6968 (2)	0.0562 (6)	
N1B1	0.57019 (12)	0.3380 (2)	0.67793 (17)	0.0317 (5)	
C61	0.41011 (13)	0.6651 (2)	0.58391 (18)	0.0283 (5)	
N1	0.33086 (11)	0.48247 (19)	0.56945 (15)	0.0266 (4)	
O3S	0.71671 (16)	−0.3041 (3)	0.7977 (2)	0.0683 (8)	
C4B	0.80457 (13)	0.2258 (2)	0.76527 (19)	0.0285 (5)	
O2S	0.81545 (12)	−0.2037 (2)	0.75528 (17)	0.0502 (6)	
C5	0.26391 (13)	0.6720 (2)	0.51419 (19)	0.0292 (5)	
H5	0.2640	0.7577	0.5061	0.035*	
C41	0.11792 (14)	0.6673 (3)	0.4435 (2)	0.0322 (5)	
C3B1	0.70266 (15)	0.3893 (3)	0.7259 (2)	0.0393 (7)	
H3B1	0.7398	0.4508	0.7388	0.047*	
C6B1	0.66448 (14)	0.1778 (2)	0.7099 (2)	0.0320 (5)	
H6B1	0.6755	0.0933	0.7131	0.038*	
C5B1	0.58987 (14)	0.2179 (3)	0.6825 (2)	0.0347 (6)	
H5B1	0.5513	0.1585	0.6664	0.042*	
C5B	0.82614 (16)	0.1080 (3)	0.7465 (2)	0.0399 (7)	
H5B	0.7892	0.0508	0.7103	0.048*	
C2B1	0.62669 (15)	0.4210 (3)	0.6999 (2)	0.0397 (6)	
H2B1	0.6142	0.5049	0.6975	0.048*	
C2	0.26343 (13)	0.4251 (2)	0.53933 (19)	0.0288 (5)	
H2	0.2633	0.3396	0.5485	0.035*	
C3B	0.86230 (16)	0.3063 (3)	0.8178 (3)	0.0486 (8)	
H3B	0.8509	0.3867	0.8318	0.058*	
O1S	0.71547 (15)	−0.3118 (3)	0.6300 (2)	0.0710 (8)	
C6B	0.90251 (15)	0.0755 (3)	0.7817 (2)	0.0372 (6)	
H6B	0.9157	−0.0042	0.7685	0.045*	
C2B	0.93727 (16)	0.2667 (3)	0.8495 (3)	0.0541 (9)	
H2B	0.9754	0.3228	0.8842	0.065*	
N1B	0.95814 (12)	0.1532 (2)	0.83358 (18)	0.0332 (5)	
O1W	0.10573 (14)	0.4228 (2)	0.27392 (19)	0.0519 (6)	
H1AW	0.0712 (16)	0.435 (4)	0.2194 (15)	0.062*	
H1BW	0.1360 (18)	0.375 (3)	0.264 (3)	0.062*	
O2W	0.43373 (12)	0.3359 (3)	0.48008 (17)	0.0513 (6)	
H2BW	0.4601 (19)	0.363 (4)	0.449 (3)	0.062*	
H2AW	0.3894 (8)	0.326 (4)	0.442 (2)	0.062*	
O21W	0.40118 (13)	0.2081 (2)	0.63974 (18)	0.0447 (5)	
H21A	0.3698 (16)	0.209 (3)	0.668 (2)	0.054*	
H21B	0.386 (2)	0.152 (2)	0.597 (2)	0.054*	
O11W	0.07172 (11)	0.3346 (2)	0.55418 (16)	0.0440 (5)	
H11A	0.0316 (11)	0.344 (4)	0.564 (3)	0.053*	
H11B	0.1089 (13)	0.329 (4)	0.6068 (15)	0.053*	
O5W	0.33986 (19)	0.0155 (3)	0.5038 (2)	0.0762 (9)	
H5AW	0.3389	0.0424	0.4493	0.091*	
H5BW	0.3658	−0.0473	0.5088	0.091*	
O4W	−0.0045 (3)	0.4496 (4)	0.0930 (3)	0.1043 (14)	
H4AW	−0.041 (2)	0.401 (5)	0.076 (5)	0.125*	
H4BW	0.014 (4)	0.418 (6)	0.054 (4)	0.125*	
O3W	0.1920 (3)	0.0473 (4)	0.5488 (3)	0.1201 (15)	
H3AW	0.1503	0.0425	0.5549	0.144*	
H3BW	0.2201	0.0916	0.5941	0.144*	
O12W	0.12484 (14)	0.2100 (2)	0.40278 (19)	0.0480 (5)	
H12A	0.1488 (19)	0.169 (3)	0.4530 (18)	0.058*	
H12B	0.1474 (19)	0.199 (4)	0.364 (2)	0.058*	
O22W	0.45581 (11)	0.4068 (2)	0.79051 (16)	0.0377 (4)	
H22A	0.4150 (10)	0.391 (3)	0.797 (3)	0.045*	
H22B	0.4929 (13)	0.371 (3)	0.831 (2)	0.045*	

### Atomic displacement parameters (Å^2^)

**Table:**

	*U*^11^	*U*^22^	*U*^33^	*U*^12^	*U*^13^	*U*^23^
Mn1	0.01547 (18)	0.0318 (2)	0.0378 (2)	−0.00094 (14)	0.00581 (15)	0.00010 (15)
Mn2	0.01635 (18)	0.0299 (2)	0.0356 (2)	0.00222 (13)	0.00679 (15)	0.00286 (15)
S3	0.0237 (3)	0.0358 (3)	0.0379 (3)	−0.0039 (2)	0.0126 (3)	−0.0078 (3)
O42	0.0181 (8)	0.0374 (10)	0.0546 (12)	0.0046 (7)	0.0087 (8)	−0.0006 (9)
O62	0.0174 (8)	0.0339 (10)	0.0491 (11)	−0.0013 (7)	0.0074 (8)	0.0057 (8)
O61	0.0278 (9)	0.0305 (10)	0.0569 (12)	−0.0052 (8)	0.0086 (9)	0.0014 (9)
C6	0.0193 (11)	0.0280 (12)	0.0280 (11)	−0.0011 (9)	0.0074 (9)	−0.0009 (9)
C4	0.0190 (11)	0.0285 (12)	0.0318 (12)	0.0028 (9)	0.0075 (9)	0.0002 (9)
O41	0.0319 (11)	0.0336 (11)	0.0861 (17)	0.0099 (9)	0.0164 (11)	0.0090 (11)
N3	0.0187 (9)	0.0279 (10)	0.0363 (11)	0.0012 (8)	0.0077 (8)	0.0009 (8)
C4B1	0.0185 (11)	0.0296 (12)	0.0322 (12)	0.0027 (9)	0.0064 (9)	0.0015 (10)
O4S	0.0382 (12)	0.0447 (13)	0.0896 (18)	0.0063 (10)	0.0277 (12)	0.0078 (12)
N1B1	0.0202 (10)	0.0348 (12)	0.0372 (11)	0.0028 (8)	0.0068 (8)	0.0011 (9)
C61	0.0205 (11)	0.0317 (13)	0.0305 (12)	−0.0036 (9)	0.0066 (9)	0.0010 (10)
N1	0.0176 (9)	0.0285 (10)	0.0313 (10)	0.0008 (8)	0.0061 (8)	0.0026 (8)
O3S	0.0730 (18)	0.0612 (16)	0.093 (2)	0.0118 (14)	0.0574 (16)	0.0194 (14)
C4B	0.0187 (11)	0.0292 (12)	0.0349 (12)	0.0019 (9)	0.0064 (9)	0.0027 (10)
O2S	0.0283 (10)	0.0618 (15)	0.0542 (13)	−0.0055 (10)	0.0072 (9)	−0.0114 (11)
C5	0.0223 (11)	0.0251 (12)	0.0387 (13)	0.0008 (9)	0.0092 (10)	0.0007 (10)
C41	0.0195 (11)	0.0347 (14)	0.0416 (14)	0.0060 (10)	0.0102 (10)	0.0029 (11)
C3B1	0.0199 (12)	0.0286 (13)	0.0628 (19)	−0.0016 (10)	0.0068 (12)	0.0024 (12)
C6B1	0.0218 (11)	0.0268 (12)	0.0450 (14)	0.0008 (9)	0.0090 (10)	−0.0015 (10)
C5B1	0.0206 (12)	0.0314 (13)	0.0500 (16)	−0.0022 (10)	0.0100 (11)	−0.0029 (11)
C5B	0.0237 (13)	0.0335 (14)	0.0549 (17)	−0.0012 (10)	0.0048 (12)	−0.0092 (12)
C2B1	0.0247 (13)	0.0270 (13)	0.0607 (18)	0.0044 (10)	0.0073 (12)	0.0021 (12)
C2	0.0192 (11)	0.0264 (12)	0.0376 (13)	0.0003 (9)	0.0064 (10)	0.0025 (10)
C3B	0.0236 (13)	0.0295 (14)	0.080 (2)	0.0028 (11)	0.0031 (14)	−0.0115 (14)
O1S	0.0519 (15)	0.090 (2)	0.0696 (17)	−0.0077 (14)	0.0206 (13)	−0.0426 (15)
C6B	0.0238 (12)	0.0311 (13)	0.0511 (16)	0.0041 (10)	0.0068 (11)	−0.0045 (12)
C2B	0.0222 (13)	0.0356 (16)	0.089 (3)	−0.0018 (11)	0.0008 (14)	−0.0155 (16)
N1B	0.0175 (10)	0.0322 (11)	0.0450 (13)	0.0025 (8)	0.0055 (9)	0.0002 (9)
O1W	0.0490 (14)	0.0593 (15)	0.0528 (13)	0.0171 (11)	0.0249 (11)	0.0136 (12)
O2W	0.0264 (10)	0.0890 (18)	0.0385 (11)	−0.0109 (11)	0.0116 (9)	−0.0063 (11)
O21W	0.0458 (12)	0.0376 (11)	0.0572 (14)	−0.0097 (9)	0.0263 (11)	−0.0072 (9)
O11W	0.0234 (9)	0.0704 (15)	0.0373 (11)	0.0081 (10)	0.0097 (8)	0.0067 (10)
O5W	0.103 (2)	0.0530 (16)	0.0651 (17)	0.0213 (15)	0.0207 (16)	−0.0019 (13)
O4W	0.121 (3)	0.105 (3)	0.066 (2)	−0.079 (3)	0.0082 (19)	0.0021 (18)
O3W	0.155 (4)	0.093 (3)	0.087 (3)	0.023 (3)	0.013 (2)	0.032 (2)
O12W	0.0500 (13)	0.0449 (13)	0.0583 (14)	0.0126 (10)	0.0305 (11)	0.0090 (10)
O22W	0.0284 (10)	0.0438 (11)	0.0395 (10)	0.0049 (8)	0.0106 (8)	0.0049 (8)

### Geometric parameters (Å, °)

**Table:**

Mn1—O12W	2.174 (2)	C5—H5	0.9300
Mn1—O11W	2.180 (2)	C3B1—C2B1	1.380 (4)
Mn1—O1W	2.187 (2)	C3B1—H3B1	0.9300
Mn1—O42	2.188 (2)	C6B1—C5B1	1.380 (3)
Mn1—N1B^i^	2.219 (2)	C6B1—H6B1	0.9300
Mn1—N3	2.272 (2)	C5B1—H5B1	0.9300
Mn2—O2W	2.154 (2)	C5B—C6B	1.381 (4)
Mn2—O21W	2.170 (2)	C5B—H5B	0.9300
Mn2—O22W	2.201 (2)	C2B1—H2B1	0.9300
Mn2—O62	2.2055 (19)	C2—H2	0.9300
Mn2—N1B1	2.214 (2)	C3B—C2B	1.380 (4)
Mn2—N1	2.282 (2)	C3B—H3B	0.9300
S3—O4S	1.454 (2)	C6B—N1B	1.333 (3)
S3—O1S	1.461 (2)	C6B—H6B	0.9300
S3—O3S	1.463 (3)	C2B—N1B	1.326 (4)
S3—O2S	1.477 (2)	C2B—H2B	0.9300
O42—C41	1.270 (3)	N1B—Mn1^ii^	2.219 (2)
O62—C61	1.268 (3)	O1W—H1AW	0.820 (5)
O61—C61	1.228 (3)	O1W—H1BW	0.818 (5)
C6—N1	1.336 (3)	O2W—H2BW	0.820 (5)
C6—C5	1.386 (3)	O2W—H2AW	0.818 (5)
C6—C61	1.528 (3)	O21W—H21A	0.820 (5)
C4—N3	1.339 (3)	O21W—H21B	0.821 (5)
C4—C5	1.384 (3)	O11W—H11A	0.820 (5)
C4—C41	1.531 (3)	O11W—H11B	0.819 (5)
O41—C41	1.217 (3)	O5W—H5AW	0.8183
N3—C2	1.330 (3)	O5W—H5BW	0.8202
C4B1—C3B1	1.389 (4)	O4W—H4AW	0.819 (5)
C4B1—C6B1	1.390 (3)	O4W—H4BW	0.820 (5)
C4B1—C4B	1.488 (3)	O3W—H3AW	0.8192
N1B1—C2B1	1.335 (3)	O3W—H3BW	0.8207
N1B1—C5B1	1.340 (3)	O12W—H12A	0.820 (5)
N1—C2	1.332 (3)	O12W—H12B	0.819 (5)
C4B—C3B	1.377 (4)	O22W—H22A	0.821 (5)
C4B—C5B	1.385 (4)	O22W—H22B	0.819 (5)
			
O12W—Mn1—O11W	86.62 (9)	C3B—C4B—C4B1	120.7 (2)
O12W—Mn1—O1W	82.31 (9)	C5B—C4B—C4B1	122.4 (2)
O11W—Mn1—O1W	168.25 (9)	C4—C5—C6	116.7 (2)
O12W—Mn1—O42	166.73 (8)	C4—C5—H5	121.6
O11W—Mn1—O42	100.38 (9)	C6—C5—H5	121.6
O1W—Mn1—O42	89.74 (9)	O41—C41—O42	127.7 (2)
O12W—Mn1—N1B^i^	96.20 (9)	O41—C41—C4	116.8 (2)
O11W—Mn1—N1B^i^	89.12 (8)	O42—C41—C4	115.5 (2)
O1W—Mn1—N1B^i^	95.96 (9)	C2B1—C3B1—C4B1	119.4 (2)
O42—Mn1—N1B^i^	95.17 (8)	C2B1—C3B1—H3B1	120.3
O12W—Mn1—N3	95.45 (9)	C4B1—C3B1—H3B1	120.3
O11W—Mn1—N3	90.24 (8)	C5B1—C6B1—C4B1	119.5 (2)
O1W—Mn1—N3	86.91 (9)	C5B1—C6B1—H6B1	120.3
O42—Mn1—N3	73.43 (7)	C4B1—C6B1—H6B1	120.3
N1B^i^—Mn1—N3	168.27 (8)	N1B1—C5B1—C6B1	123.3 (2)
O2W—Mn2—O21W	83.99 (10)	N1B1—C5B1—H5B1	118.3
O2W—Mn2—O22W	168.25 (9)	C6B1—C5B1—H5B1	118.3
O21W—Mn2—O22W	84.33 (8)	C6B—C5B—C4B	119.9 (3)
O2W—Mn2—O62	96.50 (9)	C6B—C5B—H5B	120.0
O21W—Mn2—O62	165.68 (8)	C4B—C5B—H5B	120.0
O22W—Mn2—O62	95.12 (8)	N1B1—C2B1—C3B1	123.6 (3)
O2W—Mn2—N1B1	88.22 (8)	N1B1—C2B1—H2B1	118.2
O21W—Mn2—N1B1	98.64 (9)	C3B1—C2B1—H2B1	118.2
O22W—Mn2—N1B1	92.31 (8)	N3—C2—N1	124.7 (2)
O62—Mn2—N1B1	95.68 (8)	N3—C2—H2	117.6
O2W—Mn2—N1	88.34 (8)	N1—C2—H2	117.6
O21W—Mn2—N1	93.45 (8)	C4B—C3B—C2B	119.5 (3)
O22W—Mn2—N1	93.60 (7)	C4B—C3B—H3B	120.3
O62—Mn2—N1	72.29 (7)	C2B—C3B—H3B	120.3
N1B1—Mn2—N1	167.02 (8)	N1B—C6B—C5B	123.0 (3)
O4S—S3—O1S	111.02 (17)	N1B—C6B—H6B	118.5
O4S—S3—O3S	109.49 (15)	C5B—C6B—H6B	118.5
O1S—S3—O3S	108.10 (19)	N1B—C2B—C3B	123.9 (3)
O4S—S3—O2S	111.16 (14)	N1B—C2B—H2B	118.1
O1S—S3—O2S	107.70 (14)	C3B—C2B—H2B	118.1
O3S—S3—O2S	109.30 (16)	C2B—N1B—C6B	116.8 (2)
C41—O42—Mn1	118.98 (16)	C2B—N1B—Mn1^ii^	116.33 (18)
C61—O62—Mn2	121.17 (15)	C6B—N1B—Mn1^ii^	126.39 (18)
N1—C6—C5	121.5 (2)	Mn1—O1W—H1AW	121 (3)
N1—C6—C61	115.7 (2)	Mn1—O1W—H1BW	117 (3)
C5—C6—C61	122.7 (2)	H1AW—O1W—H1BW	107 (4)
N3—C4—C5	121.6 (2)	Mn2—O2W—H2BW	124 (3)
N3—C4—C41	116.0 (2)	Mn2—O2W—H2AW	115 (3)
C5—C4—C41	122.3 (2)	H2BW—O2W—H2AW	111 (4)
C2—N3—C4	117.6 (2)	Mn2—O21W—H21A	113 (3)
C2—N3—Mn1	128.39 (17)	Mn2—O21W—H21B	131 (3)
C4—N3—Mn1	112.93 (16)	H21A—O21W—H21B	104 (4)
C3B1—C4B1—C6B1	117.3 (2)	Mn1—O11W—H11A	120 (3)
C3B1—C4B1—C4B	121.4 (2)	Mn1—O11W—H11B	123 (3)
C6B1—C4B1—C4B	121.3 (2)	H11A—O11W—H11B	113 (4)
C2B1—N1B1—C5B1	117.0 (2)	H5AW—O5W—H5BW	100.7
C2B1—N1B1—Mn2	123.17 (18)	H4AW—O4W—H4BW	93 (6)
C5B1—N1B1—Mn2	119.85 (17)	H3AW—O3W—H3BW	108.8
O61—C61—O62	126.6 (2)	Mn1—O12W—H12A	123 (3)
O61—C61—C6	118.3 (2)	Mn1—O12W—H12B	118 (3)
O62—C61—C6	115.1 (2)	H12A—O12W—H12B	105 (4)
C2—N1—C6	117.7 (2)	Mn2—O22W—H22A	112 (3)
C2—N1—Mn2	126.51 (17)	Mn2—O22W—H22B	114 (2)
C6—N1—Mn2	115.55 (15)	H22A—O22W—H22B	115 (3)
C3B—C4B—C5B	116.9 (2)		

Symmetry codes: (i) *x*−1, −*y*+1/2, *z*−1/2; (ii) *x*+1, −*y*+1/2, *z*+1/2.

### Hydrogen-bond geometry (Å, °)

**Table:**

*D*—H···*A*	*D*—H	H···*A*	*D*···*A*	*D*—H···*A*
O1W—H1AW···O4W	0.82 (1)	1.85 (1)	2.667 (5)	176 (4)
O1W—H1BW···O2S^iii^	0.82 (1)	2.11 (2)	2.891 (3)	159 (4)
O2W—H2BW···O62^iv^	0.82 (1)	1.96 (1)	2.775 (3)	172 (4)
O2W—H2AW···O1S^iii^	0.82 (1)	1.87 (1)	2.681 (3)	172 (4)
O21W—H21A···O3S^v^	0.82 (1)	1.86 (1)	2.663 (3)	166 (4)
O21W—H21B···O5W	0.82 (1)	1.97 (1)	2.782 (4)	172 (4)
O11W—H11A···O42^vi^	0.82 (1)	1.95 (1)	2.760 (3)	167 (4)
O11W—H11B···O2S^v^	0.82 (1)	1.99 (1)	2.797 (3)	168 (4)
O5W—H5AW···O4S^iii^	0.82	2.12	2.927 (4)	168
O5W—H5BW···O61^vii^	0.82	2.15	2.910 (3)	154
O4W—H4AW···O41^viii^	0.82 (1)	1.89 (1)	2.702 (4)	170 (6)
O4W—H4BW···O4W^ix^	0.82 (1)	2.48 (6)	2.908 (7)	114 (6)
O3W—H3BW···O3S^v^	0.82	1.93	2.746 (5)	178
O12W—H12A···O3W	0.82 (1)	1.85 (1)	2.656 (4)	167 (4)
O12W—H12B···O2S^iii^	0.82 (1)	2.04 (1)	2.840 (3)	166 (4)
O22W—H22A···O4S^v^	0.82 (1)	1.95 (1)	2.764 (3)	171 (3)
O22W—H22B···O61^x^	0.82 (1)	2.03 (1)	2.852 (3)	177 (3)

Symmetry codes: (iii) −*x*+1, −*y*, −*z*+1; (iv) −*x*+1, −*y*+1, −*z*+1; (v) −*x*+1, *y*+1/2, −*z*+3/2; (vi) −*x*, −*y*+1, −*z*+1; (vii) *x*, *y*−1, *z*; (viii) −*x*, *y*−1/2, −*z*+1/2; (ix) −*x*, −*y*+1, −*z*; (x) −*x*+1, *y*−1/2, −*z*+3/2.
